# Non-neurotoxic activity of Malayan krait (*Bungarus candidus*) venom from Thailand

**DOI:** 10.1186/s40409-018-0146-y

**Published:** 2018-03-09

**Authors:** Mongkon Charoenpitakchai, Kulachet Wiwatwarayos, Nattapon Jaisupa, Muhamad Rusdi Ahmad Rusmili, Supachoke Mangmool, Wayne C. Hodgson, Chetana Ruangpratheep, Lawan Chanhome, Janeyuth Chaisakul

**Affiliations:** 1 Department of Pathology, Phramongkutklao College of Medicine, Bangkok, 10400 Thailand; 2 Department of Anatomical Pathology, Army Institute of Pathology, Royal Thai Army Medical Department, Bangkok, 10400 Thailand; 3 Department of Pharmacology, Phramongkutklao College of Medicine, Bangkok, 10400 Thailand; 4 Kulliyyah of Pharmacy, International Islamic University Malaysia, Kuantan Campus, Bandar Indera Mahkota, 25200 Kuantan, Pahang Darul Makmur Malaysia; 5 Department of Pharmacology, Faculty of Pharmacy, Mahidol University, Bangkok, 10400 Thailand; 6 Monash Venom Group, Department of Pharmacology, Biomedical Discovery Institute, Monash University, Clayton, VIC 3800 Australia; 7 Queen Saovabha Memorial Institute, Thai Red Cross Society, Bangkok, 10330 Thailand

**Keywords:** Krait, *Bungarus candidus*, Myotoxicity, Nephrotoxicity, Venom, Kidney

## Abstract

**Background:**

Envenoming by kraits (genus *Bungarus*) is a medically significant issue in South Asia and Southeast Asia. Malayan krait (*Bungarus candidus*) venom is known to contain highly potent neurotoxins. In recent years, there have been reports on the non-neurotoxic activities of krait venom that include myotoxicity and nephrotoxicity. However, research on such non-neurotoxicity activities of Malayan krait venom is extremely limited. Thus, the aim of the present study was to determine the myotoxic, cytotoxic and nephrotoxic activities of *B. candidus* venoms from northeastern (BC-NE) and southern (BC-S) Thailand in experimentally envenomed rats.

**Methods:**

Rats were administered Malayan krait (BC-NE or BC-S) venom (50 μg/kg, i.m.) or 0.9% NaCl solution (50 μL, i.m.) into the right hind limb. The animals were sacrificed 3, 6 and 24 h after venom administration. The right gastrocnemius muscle and both kidneys were collected for histopathological analysis. Blood samples were also taken for determination of creatine kinase (CK) and lactate dehydrogenase (LDH) levels. The human embryonic kidney cell line (HEK-293) was used in a cell proliferation assay to determine cytotoxic activity.

**Results:**

Administration of BC-NE or BC-S venom (50 μg/kg, i.m.) caused time-dependent myotoxicity, characterized by an elevation of CK and LDH levels. Histopathological examination of skeletal muscle displayed marked muscle necrosis and myofiber disintegration 24 h following venom administration. Both Malayan krait venoms also induced extensive renal tubular injury with glomerular and interstitial congestion in rats. BC-NE and BC-S venoms (100–0.2 μg/mL) caused concentration-dependent cytotoxicity on the HEK-293 cell line. However, BC-NE venom (IC_50_ = 8 ± 1 μg/mL; at 24 h incubation; *n* = 4) was found to be significantly more cytotoxic than BC-S venom (IC_50_ = 15 ± 2 μg/mL; at 24 h incubation; *n* = 4). In addition, the PLA_2_ activity of BC-NE venom was significantly higher than that of BC-S venom.

**Conclusions:**

This study found that Malayan krait venoms from both populations possess myotoxic, cytotoxic and nephrotoxic activities. These findings may aid in clinical diagnosis and treatment of envenomed patients in the future.

## Background

A number of krait species (genus *Bungarus*) are found throughout the Indian subcontinent (including Sri Lanka and Bangladesh), and in most parts of Southeast Asia and southern China. Malayan krait (*Bungarus candidus*) is classified into category 1 of medically important venomous snakes in Indonesia (Sumatra, Java and Bali) and Thailand [1]. Previous studies have shown that phospholipase A_2_ (PLA_2_) and three-finger toxins (3FTxs) are the major components of Malayan krait venom and responsible for the neurotoxicity following envenoming [2, 3]. In addition, non-neurotoxic symptoms such as rhabdomyolysis and cardiovascular disturbances (e.g. hypertension and shock) were observed following Malayan krait envenoming in Vietnam [4]. Indeed, the mechanism behind Malayan krait envenoming-induced cardiovascular disturbance was recently demonstrated to involve a combination of vascular mediators and autonomic adaptation [5].

Myotoxicity is commonly observed following viper, sea snake and some elapid envenoming, and can be categorized as local or systemic myotoxicity [6–9]. Untreated severe myonecrosis may cause morbidity and mortality. Local myotoxicity affects tissue around the bite site while systemic myotoxicity causes rhabdomyolysis, which is associated with an increase in plasma creatine kinase (CK) and myoglobinuria [9].

Snake venom myotoxins were reported to cause myotoxicity via plasma membrane hydrolysis and depolarization [10]. These toxins have been purified from viper, sea snake, and elapid venoms e.g. Asp49 PLA_2_ myotoxin was isolated from *Bothrops asper* venom, PLA-H1 from *Hydrophis cyanocinctus* venom whereas acanmyotoxins were purified from death adder (*Acanthophis* sp.) venoms [8, 11–13].

The occurrence of rhabdomyolysis and increased serum CK levels following krait envenoming have been reported in cases involving Vietnamese *B. multicinctus* and *B. candidus*. However, these symptoms appeared to be absent in envenomed victims from other locations such as Malaysia and Thailand [14]. This variation may be due to divergence in venom composition and biological activities associated with geographic location [15, 16].

Evidence pertaining to myotoxic, cytotoxic and nephrotoxic activities following Southeast Asian krait envenoming is limited. In this study, we aim to determine the myotoxicity, nephrotoxicity and cytotoxicity of venoms collected from northeastern and southern Thailand *B. candidus* populations. These data will provide additional insights into the geographical differences between these localities and improve the capability of the healthcare workers to recognize and treat *B. candidus* envenoming.

## Methods

### Venom preparation and storage

Pooled and freeze-dried Malayan krait (*B. candidus*) venoms from specimens captured in northeast and south of Thailand were obtained from Queen Saovabha Memorial Institute (QSMI), Thai Red Cross Society, Bangkok, Thailand. The venom sample from the northeastern population (BC-NE) consisted of pooled venom from 3 specimens captured in Nakhon Ratchasima whereas, venom from 4 specimens captured in Nakhon Si Thammarat were pooled for the southern population (BC-S) venom sample. The snakes were milked by directly attaching a microhaematocrit tube to each fang, and the venom transferred to a 1.5 mL microcentrifuge. Fresh venom was frozen at − 20 °C, freeze-dried and then stored at − 20 °C prior to use. When required, the venoms were weighed and dissolved in distilled water as a stock solution (1 mg/mL) and diluted in 0.9% NaCl solution. Dissolved solutions were kept on ice during experiments.

### Protein quantification by bicinchoninic acid assay (BCA)

BCA protein assay kit (Pierce Biotechnology, USA) was used to determine venom protein content. In brief, venoms (25 μL) were loaded on to a 96-well plate in triplicate, then 200 μL reagent buffer mix was added to each well. The plate was incubated at 37 °C for 30 min, then read at 562 nm using a plate reader spectrophotometer (EnSpire® Multimode Plate Reader, PerkinElmer, USA). Protein concentration was determined from the standard curve.

### Animal ethics and care

Male Wistar rats (200–250 g) were purchased from the National Laboratory Animal Centre, Mahidol University, Salaya, Nakhon Pathom, Thailand. Rats were kept in stainless steel containers with access to food and drinking water ad libitum. Approvals for all experimental procedures were granted from the Subcommittee for Multidisciplinary Laboratory and Animal Usage of Phramongkutklao College of Medicine (Documentary Proof of Ethical Clearance no: IRBRTA 1130/2560) and Animal Care and Use Committee of Faculty of Science, Mahidol University (Documentary Proof of Ethical Clearance no: MUSC59–002-335).

### Preliminary experiment to determine venom dose

Preliminary experiments examined the effects of BC-S and BC-NE venoms, administered to three rats, in intramuscular (i.m.) doses of 50, 100 and 200 μg/kg. Venom doses ≥100 μg/kg (i.m.) resulted in the death of the rats within 6 h. Subsequently, a dose of 50 μg/kg (i.m.) was chosen for further experiments.

### Animal treatments

The animals were divided into nine groups (administration of 0.9% NaCl solution or the two venoms in three different post-injection periods – 3, 6 and 24 h). Rats (4–5 animals per experimental group and control) were anaesthetized with Zoletil® (20 mg/kg) and Xylazine® (5 mg/kg) via intraperitoneal (i.p.) route. Venoms were dissolved in 0.9% NaCl. BC-NE venom (dissolved in 50 μL 0.9% NaCl), BC-S venom (dissolved in 50 μL 0.9% NaCl) or 0.9% NaCl (control, 50 μL) was injected into the extensor muscles of the right hind limb.

Following injection of venom or saline, all animals were lightly anaesthetized using Zoletil® (20 mg/kg, i.p.) and Xylazine® (5 mg/kg, i.p.) and sacrificed prior to blood and tissue collection at 3, 6 or 24 h as per below.

### Blood collection for determination of creatine kinase, lactate dehydrogenase, creatinine, blood urea nitrogen and relevant electrolytes levels

Approximately 0.5 mL of blood was obtained by cardiac puncture and collected in MiniCollect® separation tubes at 3, 6 and 24 h post-injection of venom or 0.9% NaCl. After collection, the samples were centrifuged at 5500 rpm for 10 min. The supernatant was stored at − 20 °C for no longer than 12 h, before determination of creatine kinase (CK) and lactate dehydrogenase (LDH) levels. For early determination of creatinine, blood urea nitrogen (BUN) and relevant electrolytes levels (Na^+^, K^+^, Cl^−^ and HCO_3_^−^ levels), only blood samples at 3 h post-injection were used. All enzymes and electrolytes levels were detected with liquid assays supplied by Roche Diagnostics Corporation (USA).

### Histopathological studies

The right gastrocnemius muscle and both kidneys were removed and preserved in 10% formaldehyde before being embedded in paraffin. Embedded samples were cut and stained with hematoxylin-eosin (H&E) and/or periodic acid Schiff (PAS). Tissue examination was performed under a light microscope (Olympus BH-2, Olympus Optical Co., Japan). Areas in the slide with pathological changes due to typical myotoxicity and nephrotoxicity were photographed using an Olympus C-35 AD camera (Olympus Optical Co., Japan).

### Cell culture

The human embryonic kidney cell line (HEK-293) was purchased from the American Type Culture Collection (ATCC, USA). Cells were grown in a cell culture dish in Dulbecco’s modified Eagle’s medium supplemented with 10% fetal calf serum and 1% penicillin/streptomycin (10% DMEM), incubated at 37 °C with 5% CO_2_ until 70% confluence. Cells were lifted using trypsin, and pelleted. HEK-293 cells (100 μL) were plated into four 96-well cell culture plates at a density of 2.0 × 10^4^ cells/well. Plates were incubated at 37 °C in an atmosphere of 5% CO_2_.

### Cell proliferation assay

HEK-293 cells were grown overnight. Venom stock solutions were diluted in 10% DMEM to a final concentration of 0.2–100 μg/mL. Venom samples were added to wells in a cell culture plate in quadruplicate (100 μL/well). Culture medium control (cells and medium with no venom) and medium blanks (no cells) were run in parallel. The plates were incubated at 37 °C with 5% CO_2_ for 2, 4 or 24 h. After incubation, the medium was replaced with 200 μL of 1% DMEM and 50 μL of MTT solution (1 mg/mL). The plates were further incubated at 37 °C with 5% CO_2_ for 4 h. The medium was then removed and 100 μL of DMSO was added into each well to dissolve formazan crystals. The plate was read using a Tecan microplate reader at 570 nm. The percentage of viable cells was determined as described previously [17].

### Determination of PLA_2_ activity

PLA_2_ activity of the venoms was determined using a secretory PLA_2_ colorimetric assay kit (Cayman Chemical, USA, cat no. 765001) as previously described [18].

### Chemicals and drugs

The following chemicals were purchased from Sigma-Aldrich (USA): MTT, DMSO, eosin, 1% PAS, hydrochloric acid, Schiff’s Leuco-fuchsin solution and Mayer’s Haemalum. The following chemicals were purchased from other companies as indicated: BCA Protein Assay Kit (Pierce Biotechnology, USA) and DMEM (Thermo Fisher Scientific, USA).

### Data analysis and statistics

Plasma LDH, CK, plasma BUN, creatinine and electrolyte levels are presented as mean ± standard deviation (SD). The 95% confidence interval (95%CI) was calculated. For the cell-based assays, sigmoidal growth curves were presented as a percentage of maximal cell growth (% cell viability) versus log venom concentration and graphed using GraphPad Prism 6 (GraphPad Software Inc., USA). IC_50_ concentrations were determined to allow for a comparison of venom potency. Student’s unpaired *t*-test was performed on venom responses in the presence of venoms in different samples. Multiple comparisons were made using one-way analysis of variance (ANOVA) followed by Bonferroni’s multiple comparison test.

## Results

### Plasma electrolyte, BUN and creatinine levels following 3 h of envenomation

Blood samples were collected following 3 h of administration of venoms to evaluate their early effects on relevant electrolytes and enzymes. Administration of BC-NE or BC-S venom (50 μg/kg, i.m.) significantly increased BUN and potassium levels over a 3-h period when compared with 0.9% NaCl (*n* = 4, *p* < 0.05, Student’s unpaired *t*-test, Table 1). A significant increase in creatinine levels was observed following administration of BC-S venom, but not BC-NE venom. BC-S venom also attenuated plasma Na^+^ concentration when compared to controls (*n* = 4, *p* < 0.05, Student’s unpaired *t*-test, Table 1). There was no significant change in plasma chloride and bicarbonate levels over the 3 h period following administration of either venom.

**Table 1:** Plasma electrolyte, BUN and creatinine levels 3 h following administration of Malayan krait venoms (50 μg/kg, i.m.) from different populations or saline (50 μL, i.m.)

	BUN (mg/dL)	Creatinine (mg/dL)	Na^+^ (mEq/L)	K^+^ (mEq/L)	Cl^−^ (mEq/L)	HCO_3_^−^ (mEq/L)
Saline/Control	24.7 ± 1	0.25 ± 0	147.3 ± 1	3.8 ± 0	94.1 ± 1	22.7 ± 0
BC-NE	40.1 ± 1*	0.45 ± 0	135.8 ± 5	7.9 ± 1*	90.7 ± 4	23.7 ± 0
BC-S	44.3 ± 3*	0.53 ± 0*	132.5 ± 4*	10.3 ± 2*	89.5 ± 3	18.5 ± 2

*p < 0.05 is significantly different from control (Student’s unpaired *t*-test), *n* = 4

### Plasma LDH levels

**Fig. 1:**
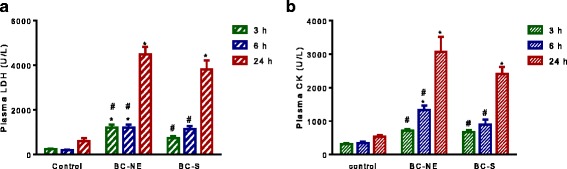
(**a**) Plasma lactate dehydrogenase (LDH) and (**b**) plasma creatine kinase (CK) levels at 3, 6 and 24 h following intramuscular (i.m.) administration of saline/control (50 μL), BC-S venom (50 μg/kg) or BC-NE venom (50 μg/kg) (*n* = 4–5). *p < 0.05 is significantly different from control at the same incubation period (Student’s unpaired *t*-test). ^#^*p* < 0.05 is significantly different from 24 h (one-way ANOVA)

BC-S and BC-NE (50 μg/kg, i.m.) venoms caused a time-dependent increase in plasma LDH levels at 3, 6, and 24 h compared to control. A marked elevation in LDH levels (> 3500 U/L) was seen following venom administration at 24 h (*n* = 4–5, *p* < 0.05, one-way ANOVA, followed by Bonferroni *t*-test, Fig. 1a).

### Plasma CK levels

Venoms (50 μg/kg, i.m.) from both localities significantly increased plasma CK levels 3 and 6 h after administration compared to control. At 24 h, both venoms significantly increased plasma CK levels (> 2500 U/L) compared to the levels at the 3 and 6 h time points (*n* = 4–5, *p* < 0.05, one-way ANOVA, followed by Bonferroni *t*-test, Fig. 1b).

### Histopathological studies

**Fig. 2:**
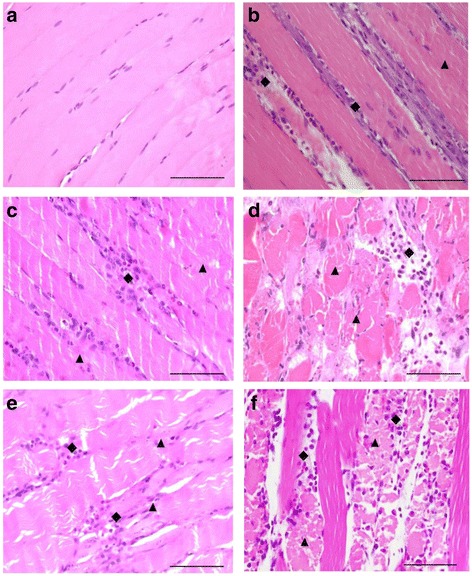
Morphological changes (H&E stain, 400× magnification) of rat gastrocnemius muscle following intramuscular (i.m.) administration of (**a**) vehicle control (normal saline 50 μL), BC-NE (50 μg/kg) venom for (**b**) 3 h, (**c**) 6 h and (**d**) 24 h, or administration of BC-S venom for (**e**) 6 h and (**f**) 24 h. Diamond shapes indicate neutrophilic infiltrate, triangles indicate disintegrating myofibers. Scale = 50 μm

Skeletal muscle displayed a minor degree of myofiber disintegration and neutrophilic infiltration 3 and 6 h following administration of venoms. Both venoms caused generalized muscle necrosis and a high degree of disintegrating muscle fiber with mononuclear infiltrate 24 h after venom administration (Fig. 2).

**Fig. 3:**
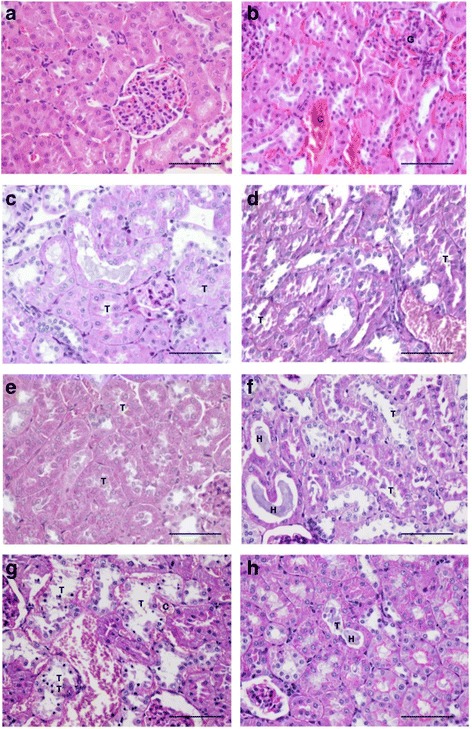
Morphological changes (H&E stain; 400× magnification) of rat kidneys following intramuscular administration of (**a**) vehicle control and (**b**) BC-NE venom for 3 h. Morphological changes (PAS stain; 400× magnification) of rat kidneys following intramuscular (i.m.) administration of BC-NE venom for (**c**) 3 h, (**e**) 6 h and (**g**) 24 h. Effect of BC-S venom on the morphological changes of rat kidneys following i.m. administration after (**d**) 3 h, (**f**) 6 h and (**h**) 24 h. Tubular injury is represented by "T". "H" represents hyaline cast. Interstitial congestion is represented by "C". "G" indicates glomerular congestion. Scale = 50 μm

Rat kidneys exhibited mild to moderate morphological changes at the 3 and 6 h time points following BC-NE or BC-S administration (50 μg/kg; i.m.). These changes were characterized by the presence of hyaline cast, dilatation of renal capillary, diffuse or focal glomeruli and/or interstitial vessels congestion (Fig. 3b) and tubular injury (Fig. 3c–e) with loss of brush border. Severe congestion with hemorrhage was observed in glomeruli and interstitial vessels 24 h following the injection of BC-NE venom (50 μg/kg; i.m.) (Fig. 3g). Administration of BC-S venom (50 μg/kg; i.m.) also caused diffuse tubular injury with loss of brush border and the presence of hyaline cast after 6 and 24 h (Fig. 3f and h, respectively).

### Cell viability assay: venom concentration-response curve

**Fig. 4:**
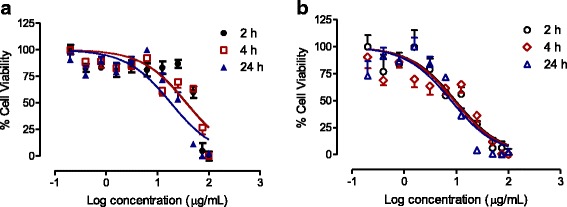
Sigmoidal growth curves for (**a**) BC-S and (**b**) BC-NE venoms (100–0.2 μg/mL) after 2, 4 or 24 h of incubation in HEK-293 cells (*n* = 4)

Incubation of HEK-293 cells with BC-S or BC-NE venoms (100–0.2 μg/mL) caused concentration-dependent inhibition of cell viability (Fig. 4a and b). The IC_50_ value of BC-NE venom was significantly lower than that of BC-S venom following incubation for 2–24 h (*p* < 0.05, Student’s unpaired *t*-test, Table 2) indicating that BC-NE venom was significantly more cytotoxic compared to BC-S venom.

**Table 2:** IC_50_ values for BC-S and BC-NE venoms (*n* = 4) on HEK-293 cell line

	IC_50_ concentration (μg/mL)	
Incubation time	BC-S	BC-NE
2 h	42 ± 7	15 ± 2^*^
4 h	26 ± 2^#^	15 ± 3^*^
24 h	15 ± 2^#^	8 ± 1^*^

*p < 0.05 is significantly different from BC-S venom at the same incubation time (Student’s unpaired *t*-test)

^#^*p* < 0.05 is significantly different from 2 h incubation (one-way ANOVA)

### Time-dependent effect of venoms incubated with HEK-293 cells

There was no significant difference among the concentration-response curves of BC-NE (100–0.2 μg/mL) venom for any time period (2-, 4- and 24-h). However, BC-S venom (100–0.2 μg/mL) caused significant time-dependent cytotoxic effects on the HEK-293 cell line following 4 to 24 h incubation (Table 2).

### Phospholipase A_2_ activity

PLA_2_ activity for BC-S and BC-NE venoms were 573 ± 23 and 1558 ± 178 μmol/min/mg (*n* = 3), respectively. The PLA_2_ activity for the positive control, i.e. bee venom, was 505 ± 22 μmol/min/mg (*n* = 3).

## Discussion

Systemic myotoxicity is observed following envenoming by sea snake, some viperids and elapids [7, 19, 20]. Clinical outcomes following systemic venom-induced myotoxicity include widespread muscle injury with associated myalgia, elevation of plasma CK level, myoglobinuria and hyperkalemia due to extensive muscle cell damage [9, 21]. Previous works have shown that systemic envenoming by *Bungarus niger* could lead to neurotoxic envenoming, myoglobinuria and acute renal failure [22, 23]. *B. candidus* envenomed patients in Vietnam were reported to display symptoms of rhabdomyolysis and serum CK level elevation [4]. Although severe generalized myalgia and acute renal injury following envenoming by Malayan krait have been anecdotally reported in Thailand [4, 24], studies regarding myotoxicity and nephrotoxicity have yet to be carried out.

The determination of CK, LDH and relevant electrolyte concentrations are important to the diagnosis of nephrotoxicity and muscular damage. Our data showed that Malayan krait venoms from northeastern and southern Thailand caused significant increases in serum CK and LDH levels following injection. This result is consistent with a Vietnamese case report that showed a rise in CK level in victims after systemic envenoming [4].

In the present study, CK elevation did not reach maximum value within 6 h as seen for viperid myotoxins or coral snake venoms [9, 25]. This result is in agreement with a recent study indicating no significant elevation of CK level following Sri Lankan Russell’s viper envenoming at the 6 h time point [19]. The variation in CK levels following venom administration could be attributed to differences in either the pharmacokinetics of venom distribution or method of administration. Indeed, administration of venom via intramuscular or subcutaneous routes may cause a slower rise in venom concentration, resulting in a delayed increase in CK level [6]. In contrast, intravenous administration induced a rapid elevation in CK level due to the 100% bioavailability of venom compared to intramuscular or subcutaneous ones [6].

Early detection of electrolytes is necessary to predict myotoxicity and rhabdomyolysis in envenomed patients. In the present study, injection of Malayan krait venoms from either locality caused elevation of serum BUN, creatinine and potassium levels after 3 h, suggesting that acute kidney injury can be detected in the early stage of envenoming. Interestingly, BC-S venom significantly decreased plasma sodium levels, which suggests hyponatremia. These laboratory data are consistent with previous case reports [4, 20]. In our study, relevant electrolytes were determined following 3 h of envenoming. This would be of benefit for the diagnosis of early myotoxicity and nephrotoxicity, as many previous reports displayed the changes in electrolyte in later stages (i.e. after 6 h of envenoming) [4, 23].

Snake venom-induced skeletal muscle damage is characterized by hypercontraction of myofilaments, disruption of the plasma membrane, and tissue necrosis including release of CK [10]. Renal damage can be induced by direct and indirect myotoxic effects of toxins. The indirect effects cause nephrons to be overloaded by degraded proteins, including myoglobin from decayed tissue tubules, which result in secondary acute kidney injury [8, 26, 27]; whereas direct effects cause damage to the kidney cells due to cytotoxicity [28, 29].

In this work, we found that Malayan krait venoms from southern and northeastern populations induced time-dependent myotoxic and nephrotoxic activities. A high degree of myofiber disintegration in skeletal tissues was seen 24 h following venom administration and this correlates with the increase in plasma CK levels. A lower degree of muscle necrosis was detected as early as 3 h after venom administration. In the kidneys, the presence of hyaline cast was seen in renal tubules after 3 h venom administration. Similar renal morphologic changes have also been found in tissues exposed to the venoms of some Russell’s vipers, *Micrurus* species and other terrestrial elapids [25, 30–32].

A range of cytotoxic components in snake venoms may contribute to the severity and development of myotoxicity. Snake myotoxins can be classified into three different groups [33]:

- ‘small myotoxins’ from rattlesnake venoms such as crotamine from the venom of *Crotalus durissus terrificus* [34, 35];
- ‘cardiotoxins’, the purified toxins from cobra venom that belong to the 3FTx family [36, 37];
- PLA_2_s, the most abundant myotoxic components in elapid and viperid venoms [38].

Using proteomic techniques, 3FTx and PLA_2_ were found to be the main protein components of Malayan krait venoms [2]. However, Malayan krait myotoxins have not been isolated and characterized. Therefore, the effects of these myotoxins are unknown.

Local muscle necrosis and tissue gangrene are rarely observed following Malayan krait envenoming [4]. However, we found that both venoms displayed cytotoxicity indicating the presence of potent cytotoxins in the venom of this species. The cytotoxic effects of Malayan krait venom were determined using human embryonic kidney (HEK-293) cells. Snake venom cytotoxicity has been demonstrated using various cell lines including rat skeletal muscle, aortic smooth muscle and mammalian kidney cells [39, 40]. The cell-based assay is a practical model for the determination of cytotoxicity of snake venoms and may be used together with isolated tissue preparations to pharmacologically characterize animal venoms [39]. However, it may not reflect venom-mediated effects in vivo [41].

The comparison of IC_50_ at all incubation periods showed that BC-NE venom was significantly more cytotoxic than BC-S venom, which indicates a difference in venom composition and potency. However, the use of different cell lines (i.e. skeletal muscle cells or cardiomyocytes) might indicate different degrees of cytotoxicity [39]. In addition, BC-NE venom was found to have significantly higher PLA_2_ activity than BC-S venom, which may be due to geographical variation.

## Conclusions

In conclusion, we have demonstrated that Malayan krait venoms from two different locations in Thailand display myotoxicity and nephrotoxicity in an animal model with histological evaluation of tissue. We also have shown that BC-S and BC-NE venoms have significant cytotoxic effects on human embryonic kidney cells. BC-NE venom was found to be more cytotoxic than BC-S venom, but significant differences in myotoxicity and nephrotoxicity were not observed.

## Abbreviations

3FTx: three-finger toxin
95%CI: 95% confidence interval
ANOVA: analysis of variance
ATCC: American Type Culture Collection
BCA: bicinchoninic acid assay
BC-NE: *Bungarus candidus* from northeastern Thailand
BC-S: *Bungarus candidus* from southern Thailand
BUN: blood urine nitrogen
CK: creatine kinase
DMEM: Dulbecco’s modified Eagle’s medium
DMSO: dimethyl sulfoxide
h: hour
H&E: hematoxylin and eosin
i.m.: intramuscular
i.p.: intraperitoneal
IC_50_: the half maximal inhibitory concentration
LDH: lactate dehydrogenase
MTT: methylthiazolyldiphenyl-tetrazolium bromide
PAS: Periodic acid Schiff
PLA_2_: phospholipases A_2_
SD: standard deviation
